# Whitefly Cryptic Species in Nigeria–Cameroon Border Regions and Their Acquired Cassava Mosaic Begomoviruses

**DOI:** 10.3390/insects17100979

**Published:** 2026-09-22

**Authors:** Oghenevwairhe P. Efekemo, Olabode A. Onile-ere, Isaac O. Abegunde, Folashade T. Otitolaye, Patrick Kenfack-Fogang, Wenceslas Yana, Oumar Doungous, Oghenetega C. Ogbe, Chukwuemeka K. Nkere, Ibrahim J. Yusuf, Ibrahim U. Mohammed, Justin S. Pita, Titus Alicai, Angela O. Eni

**Affiliations:** 1 Department of Biological Sciences, Covenant University, Km. 10 Idiroko Road, Ota 12212, Ogun State, Nigeriafolashadeotitolaye.fo@gmail.com (F.T.O.); 2 Central and West African Virus Epidemiology (WAVE) Program, Covenant University Hub, Km. 10 Idiroko Road, Ota P.M.B. 1023, Ogun State, Nigeria; 3 Centre of Excellence in Agricultural Development and Sustainable Environment, Federal University of Agriculture Abeokuta (FUNAAB), Alabata Road, Abeokuta P.M.B. 2240, Ogun State, Nigeria; 4 Central Entomology Laboratory, Mbalmayo Regional Research Centre, Institute of Agricultural Research for Development, Yaounde P.O. Box 2067, Cameroon; pkenfackfogang@yahoo.com; 5 Department of Zoology, Faculty of Sciences, University of Bamenda, Bambili P.O. Box 39, Cameroon; 6 Central and West African Virus Epidemiology (WAVE) Program, Biotechnology Laboratory, Ekona Regional Research Centre, Institute of Agricultural Research for Development, Buea P.O. Box 25, Cameroon; doungous.oumar@wave-center.org; 7 National Root Crops Research Institute (NRCRI), Umuahia-Ikot Ekpene Federal Road, Umudike P.M.B. 7006, Abia State, Nigeriankerechukwuemeka@yahoo.com (C.K.N.); 8 Department of Crop Science, Faculty of Agriculture, Abdullahi Fodio University of Science and Technology, Aliero (AFUSTA), Sokoto-Jega Road, Aliero P.M.B. 1144, Kebbi State, Nigeria; ibrahimyusuf090@gmail.com (I.J.Y.); iumohammed74@gmail.com (I.U.M.); 9 Regional Center of Excellence for Transboundary Plant Pathogens, Central and West African Virus Epidemiology (WAVE), Pôle Scientifique et d’Innovation, Université Félix Houphouët-Boigny, Abidjan BPV 34, Côte d’Ivoire; 10 National Agricultural Research Organization (NARO), National Crops Resources Research Institute, Kampala P.O. Box 7084, Uganda

**Keywords:** *Bemisia tabaci* cryptic species, *Bemisia afer*, mtCOI, characterization, phylogenetic analysis, whitefly surveillance, cross-border epidemiology

## Abstract

The whitefly vector remains a key factor in the continued prevalence of Cassava Mosaic Disease (CMD) and the threat of Cassava Brown Streak Disease (CBSD) to unaffected regions in Central and West Africa. Cross-border surveillance is critical because borders serve as the first entry point for pests and pathogens. This study identified the whitefly genotype groups prevalent in cassava-producing locations along the Nigeria–Cameroon border, and their associated Cassava mosaic begomovirus (CMB) strains. Mitochondrial cytochrome oxidase I (mtCOI) gene sequencing (97 sequences), sequence alignment, and phylogenetic analysis revealed several *Bemisia tabaci* (Gennadius) genotypes alongside *Bemisia afer* (Priesner & Hosny). *B. tabaci* sub-Saharan Africa 1-subgroup 1 (SSA1-SG1), SSA2, SSA3, and Mediterranean (MED) genotypes were recorded in Nigeria and Cameroon. MED and SSA3 genotypes were predominant in the North–East and South–South regions of Nigeria, respectively, while SSA2 and SSA3 were the predominant genotypes on the corresponding side of the Cameroon border. CMB-specific PCR amplification revealed the presence of African cassava mosaic virus (ACMV) and East African cassava mosaic virus (EACMV) in several *B. tabaci* genotypes and in *B. afer*. These findings demonstrate a distinct vector distribution pattern across the Nigeria–Cameroon border and emphasize the need for continued cross-border cassava virus surveillance.

## Simple Summary

Cassava is an important food crop for millions of people in sub-Saharan Africa, but its production is threatened by viral diseases, especially Cassava Mosaic Disease (CMD) and Cassava Brown Streak Disease (CBSD). These diseases are transmitted by whitefly vectors which differ in virus transmission efficiency, host preferences, and insecticide susceptibility. Whiteflies can facilitate the spread of cassava viruses across borders through natural dispersal and the movement of infested cassava planting materials. Given the growing concern about the spread of CBSD from Central to West Africa, understanding the whitefly distribution, especially around the most important border between the Central and West African cassava production zone, would inform pre-CBSD incursion preparedness activities and aid the management of cassava virus diseases in the region. This study assessed the whitefly populations in the border regions of Nigeria and Cameroon as these two countries are pivotal to the geographical expansion of CMD and incursion of CBSD to West Africa. The results obtained emphasized the need for regular whitefly and virus surveillance, particularly in cross-border zones, to prevent and manage the spread of more virulent virus strains between Central and West Africa.

## 1. Introduction

Cassava (*Manihot esculenta* Crantz) is an important staple crop in sub-Saharan Africa, where it provides an affordable source of daily calorie intake for over 500 million people [1,2,3]. Cassava production is however constrained by several viral diseases, particularly the Cassava Mosaic Disease (CMD) and Cassava Brown Streak Disease (CBSD) [4,5,6]. CMD is caused by Cassava mosaic begomoviruses (CMBs), which belong to the genus *Begomovirus* within the family *Geminiviridae* and have been endemic to Africa since the early 20th century [7]. In Africa, the most predominant CMBs are African cassava mosaic virus (ACMV) and East African cassava mosaic virus (EACMV), including its virulent Uganda variant (EACMV-UG) [7]. CMD is responsible for substantial tuber yield losses, often exceeding 70% under severe infection conditions [7,8]. Similarly, CBSD caused by Cassava brown streak ipomoviruses (CBSIs), belonging to the genus *Ipomovirus* (family *Potyviridae*), remained endemic in East Africa until a major CBSD outbreak occurred in 2004 that led to the expansion of CBSD to other countries across East and Central Africa [5]. Recent reports indicate that CBSD continues to spread westwards, increasing the threat of CBSD in unaffected regions in Central and West Africa [9,10,11]. The continued prevalence of CMD in Africa and the ever-increasing threat of CBSD suggest the need to develop sustainable disease management strategies. Although CMD and CBSD are propagated through the use of virus-infected stem cuttings, CMD and CBSD outbreaks in East Africa were closely associated with high populations of whitefly vectors that transmit these viruses [4].

Epidemiological surveillance, especially for whitefly presence and changes in populations and species types, particularly in border regions, is a vital component of cassava viral disease management in cassava-producing areas. This is because infected plant materials, vectors, and pathogens may be accidentally or intentionally exchanged across border regions [12]. Border regions are often under-sampled and under-monitored despite being crucial points of entry for invasive species or novel pathogens/virus strains. With Nigeria ranking as the world’s largest cassava producer, and Cameroon serving as the gateway country linking Central Africa and West Africa, a coordinated surveillance strategy across these countries is essential for early detection and containment of emerging vector-virus threats [13,14].

The whitefly vector—*Bemisia tabaci* (Gennadius) (Hemiptera: Aleyrodidae) acts as the primary whitefly species involved in the transmission of CMBs and CBSIs [12]. *B. tabaci* transmits CMBs in a persistent manner and CBSIs in a semi-persistent manner [15]. In addition to its role in virus transmission, *B. tabaci* is known as a highly destructive cassava pest. It causes damage to the cassava plant by directly feeding on phloem sap, and indirectly by excreting honeydew onto the surface of the leaves. The honeydew acts as a substrate for the growth of sooty mold fungi (*Capnodium* spp.) on the surfaces of leaves interfering with photosynthesis and tuber yield [15,16]. Apart from *B. tabaci*, other whitefly species have been identified on cassava plants including *B. afer*, *Aleurodicus dispersus* (Russell), *Trialeurodes vaporariorum* (Westwood), and *Paraleyrodes bondari* (Peracchi) [17].

*B. tabaci* is regarded as a complex group made up of at least 40 morphologically indistinguishable species [18,19]. The initial classification of *B. tabaci* genotype groups A and B was based on biological traits, such as esterase profile and host range [20]. Later studies revealed that these traits were not reliable for classification because they showed within-group variability or were shared between genotype groups [21]. Several techniques have been used to study the phylogenetic properties of *B. tabaci* genotype groups, including protein allozyme electrophoresis, iso-enzyme electrophoresis, RAPDs-PCR, SCAR, CAPS, mitochondrial cytochrome oxidase 1 (mtCOI) sequencing, mitochondrial 16s, AFLP, and microsatellites, as observed in numerous studies [17,22,23,24]. The mtCOI gene sequence is the most commonly used gene to propose putative delimitation boundaries within the *B. tabaci* complex because it is a highly variable region [17,25]. There are 11 known *B. tabaci* genotype groups, namely Asia I (H, M), Asia II 5 (G), Asia II 1 (K), New World (A, D), Mediterranean (J, L), Middle East–Asia Minor (B, B2), and sub-Saharan Africa (E) [18]. Some of these groups were then sub-divided into smaller clades. For example, the Middle East–Asia Minor (MEAM) group consists of two genotype groups (MEAM 1 and MEAM 2), the Mediterranean (MED) group consists of four genotype groups (Q1, Q2, Q3, and ASL), and the sub-Saharan Africa (SSA) group, the most genetically diverse, consists of 13 known genotype groups; SSA 1-13 [15,19,26,27]. Of the SSA genotype groups, SSA1-SSA5 have been found colonizing cassava in sub-Saharan Africa [4]. Recent studies have identified more mtCO1 sequence divergence among the SSA1; hence, the group has been sub-divided into five subgroups; SSA1 subgroup 1 (SSA1-SG1), SSA1-SG2, SSA1-SG3, SSA1-SG4, and SSA1-SG5 [4].

Members of the *B. tabaci* complex differ in terms of their host-plant range, invasiveness, susceptibility to insecticides and natural enemies, fecundity, and vector competency [18]. For example, SSA2 was reported as the predominant whitefly genotype during the EACMV-UG-associated CMD pandemic that originated in Uganda in the late 1980s, and its abundance was closely associated with the geographic spread of EACMV-UG into neighboring countries by the early 2000s [28]. SSA1-SG1 has been widely reported throughout East and West Africa and is strongly associated with the spread of CMD [29]. In contrast, although not a major cassava-colonizing whitefly in Africa, MED is one of the most invasive members of the *B. tabaci* complex because of its broad host range, high reproductive capacity, adaptability to diverse host plants, and widespread insecticide resistance [22,30]. Hence, accurate identification of whitefly genotype groups is essential not only for understanding the epidemiology of CMD and CBSD but also for guiding effective control strategies.

In Nigeria, extensive whitefly genetic diversity studies have been conducted in North Central [31], South–West [21,31,32], South–East [33], and South–South zones [33]. However, there is still limited information about the distribution and genetic diversity of whitefly genotype groups in other regions of Nigeria, particularly the North–East zone, which includes states that share borders with Cameroon. Similarly, whitefly genetic studies have been conducted in Cameroon [21,34]; however, among the five regions bordering Nigeria, only the South–West region was covered in the study by Kepngop et al. [34]. Therefore, the objectives of this study are to identify the whitefly genotype group in the Nigeria–Cameroon border regions and to assess the presence of CMBs in the whitefly genotype groups identified. A clear understanding of whitefly genotype groups present in cassava fields, especially around this important border connecting the Central and West African cassava production zones, would be invaluable for disease management and preparedness planning.

## 2. Materials and Methods

### 2.1. Study Area and Sample Collection

The whiteflies used for this study were obtained from ethanol-preserved samples collected during routine epidemiological surveys conducted by the Central and West African Virus Epidemiology (WAVE) program in 2020 and 2022 [31,35,36]. The samples were collected from cassava farms in the border regions of Nigeria and Cameroon. The Nigerian samples were from cassava fields in Taraba, Adamawa, Borno, Benue, and Cross River States, while the Cameroon samples were from cassava farms in Far North, North, Adamawa, North–West, and South–West regions (Figure 1).

### 2.2. Whitefly DNA Extraction

Whitefly DNA was extracted following the Chelex DNA extraction method described by Mugerwa et al. [37]. From each sample tube, one adult whitefly was randomly selected, placed on a Parafilm, and allowed to dry for a few seconds. The whitefly was placed in a 1.5 mL microcentrifuge tube containing 50 μL of 10% (*w*/*v*) Chelex 100 solution (Sigma-Aldrich, St. Louis, MO, USA), crushed using a sterile plastic rod, and then vortexed using the vortex mixer (Cole Parmer, Vernon Hills, IL, USA) until a clear homogenized mixture was obtained. The mixture was incubated in a water bath (Cole Parmer, Vernon Hills, IL, USA) at 56 °C for 20 min and further incubated at 100 °C for 5 min. After the incubation period, the mixture was centrifuged using a microcentrifuge (Labnet, Edison, NJ, USA) at 13,500× *g* for 5 min at room temperature, and then the supernatant was collected and stored in a −20 °C freezer (LG, Seoul, Republic of Korea) until required.

### 2.3. Mitochondrial COI (mtCOI) Amplification, Sequencing, and Phylogenetic Analysis

The amplification of the mitochondrial cytochrome C oxidase 1 (mtCO1) gene of each whitefly was achieved by PCR using the primer pair 2195Bt and C012/Bt-sh2 (Table 1) designed by Mugerwa et al. [37]. The PCR was prepared to a final volume of 50 µL, containing 36.5 µL of sterile distilled water, 5 µL of 10× PCR buffer, 4 µL of 2.5 mM dNTPs, 1 µL of 10 µM of each primer, 2 μL of extracted whitefly DNA, and 0.5 µL of 1 U Taq DNA polymerase (TransGen Biotech, Beijing, China). Reaction mixtures were amplified in a Bio-Rad 96-Well T-100 Thermal Cycler (Bio-Rad, Hercules, CA, USA) using the following PCR regime: an initial denaturation step at 94 °C for 2 min; followed by 35 cycles of denaturation at 94 °C for 20 s; hybridization at 52 °C for 30 s; extension at 72 °C for one minute; and then a final extension step at 72 °C for 10 min. All PCR amplifications included appropriate positive controls (known whitefly DNA template) and negative controls (replacing template DNA with sterile distilled water) to monitor for potential contamination and false positives. A portion (2 µL) of the amplified products was separated using 1% (*w*/*v*) agarose gel electrophoresis, stained with ethidium bromide solution (10 mg/mL; Invitrogen, Thermo Fisher Scientific, Waltham, MA, USA) and then visualized using a UV gel documentation system (UV light 302 nm) (Thomas Scientific, Swedesboro, NJ, USA). Amplified PCR products with the expected band size were then selected and purified using the Easytaq DNA purification kit (TransGen Biotech, Beijing, China) following the manufacturer’s instructions and sequenced using the Sanger method (Inqaba Biotec West Africa Ltd, Ibadan, Oyo State, Nigeria).

The sequences produced were manually edited and compared against a manually curated database of representative whitefly genotype groups using the standalone BLAST version 2.13.0+ for Windows. A 3.5% divergence limit was used as the threshold for genotype groups delimitation [27] while, within SSA1 genotype groups, subgroups were designated based on >0.6% divergence in partial mtCO1 sequences as recommended by Mugerwa et al. [38] and Legg et al. [13]. Whitefly genotype groups distribution maps were generated based on the field GPS data collected during the surveys using the sf package in R version 4.2.2.

To assess relatedness between the samples from this study and those from previously published studies, representative sequences across major whitefly genotypes were obtained from Boykin et al.’s reference dataset [26], alongside representative sequences from the current study. Then, maximum-likelihood phylogenetic trees were inferred in IQ-TREE 2 [39] using ModelFinder Plus module for nucleotide-substitution model selection and 1000 ultrafast bootstrap replicates, with bootstrap support values mapped to internal nodes.

### 2.4. Polymerase Chain Reaction (PCR) Amplification for CMBs Detection

The DNA extracted from the whiteflies was screened for the presence of CMBs using the CMB specific detection primers in Table 1. The PCR assay was carried out using the protocol described by Matic et al. [40]. The PCR composition was as follows: 1.25 μL of PCR buffer (10×), 1 µL of 2.5 mM dNTPs, 0.25 μL of 10 μM of each primer, 0.125 µL of 1 U Taq DNA Polymerase, 2 μL of extracted whitefly DNA, and sterile distilled water to a final volume of 12.5 μL. The general reaction cycles were an initial denaturation at 94 °C for 2 min followed by 30 cycles of denaturation at 94 °C for 1 min, annealing at 55 °C for 1 min, extension at 72 °C for 1 min, and a final extension of 72 °C for 10 min. All PCR amplifications included appropriate positive controls (known CMBs DNA template) and negative controls (replacing template DNA with sterile distilled water) to monitor for potential contamination and false positives. Due to the limited DNA template quantity recovered per individual whitefly sample, reactions were carried out without replicates. The amplified PCR products were resolved on a 1% agarose gel stained with ethidium bromide (10 mg/mL) alongside a 1 kbp plus DNA ladder using a gel documentation system at 100 V for about 40 min. The gel was visualized under UV light using a Bench top UV transilluminator (UVP, LLC Analytik Jena US LLC, Upland, CA, USA).

**Table 1:** Primer pairs used for mtCO1 gene amplification and CMBs detection.

	Primer Name	Primer Sequences (5′ to 3′)	Target Region	Size	Reference
1	2195Bt	TGRTTTTTTGGTCATCCRGAAGT	mtCO1	867 bp	[37]
	C012/Bt-sh2	TTTACTGCACTTTCTGCC			
2	ACMVBF	TCGGGAGTGATACATGCGAAGGC	ACMV DNA-B (BV1/BC1)	628 bp	[40]
	ACMVBR	GGCTACACCAGCTACCTGAAGCT			
3	JSP 001	ATGTCGAAGCGACCAGGAGAT	ACMV DNA-A (CP)	783 bp	[41]
	JSP 002	TGTTTATTAATTGCCAATACT			
4	JSP 001	ATGTCGAAGCGACCAGGAGAT	EACMV DNA-A (CP)	780 bp	[41]
	JSP 003	CCTTTATTAATTTGTCACTGC			
5	CMBRepF	CRT CAA TGA CGT TGT ACC A	EACMV DNA-A (AC1)	650 bp	[42]
	EACMVRepR	GGT TTG CAG AGA ACT ACA TC			
6	ACMV21for	GCAGTGATGAGTTCCCCGGTGCG	DNA-A (AC3-AC2-AC1) of ACMV, EACMV, EACMCV, EACMKV, EACMMV, EACMZV and SACMV	552 bp	[40]
	ACMV21rev	ATTCCGCTGCGCGGCCATGGAGACC			

## 3. Results

### 3.1. Distribution of Whitefly Genotype Groups

A total of 97 high-quality sequences (Nigeria-52, Cameroon-45) were obtained and used for subsequent phylogenetic analyses. The identified sequences were submitted to the GenBank database in NCBI, with accession numbers from PZ493163-PZ493174; PZ493176-PZ493210; PZ493229-PZ493273; OR807575; OR807577; and OR807579-OR807581. Following comparison with reference mtCOI sequence from GenBank, two whitefly species were identified—*B. tabaci* and *B. afer*. Based on the 3.5% divergence threshold criterion, sequences classified as *B. tabaci* samples were placed into five genotype groups: SSA1, SSA2, SSA3, SSA4, and MED. The occurrence of the identified whitefly genotype groups varied across each surveyed country.

#### 3.1.1. Nigeria

In Nigeria, *B. tabaci* was the only whitefly species identified in cassava fields in the five border states. A total of four *B. tabaci* genotype groups were identified; SSA1, SSA2, SSA3, and MED (Figure 2). The Nigerian whitefly sequences from this study had between 96.69 and 100% nucleotide (nt) similarity to previously published sequences in the GenBank (Table 2). The proportion of each genotype group varied widely across the surveyed states. The MED was the predominant genotype group, accounting for 55.8% of the total whitefly isolates sequenced, followed by SSA3 (26.9%), SSA2 (11.5%) and SSA1 (5.7%). The distribution of each genotype also varied across surveyed states with MED and SSA2 occurring in Adamawa, Taraba, and Borno. SSA3 was found in Benue and Cross River; and SSA1 (SG1 and SG3) was identified only in Cross River. Benue state had the least whitefly diversity as SSA3 was the only whitefly genotype found in Benue state, while other states had at least two whitefly genotypes.

#### 3.1.2. Cameroon

Most of the isolates from Cameroon were *B. tabaci* (91.1%) while a small proportion were classified as *B. afer* (8.9%). A total of five *B. tabaci* genotype groups were observed; SSA1, SSA2, SSA3, SSA4, and MED (Figure 3). The Cameroon whitefly sequences from this study had between 98.5 and 100% nucleotide (nt) similarity to previously published sequences in the GenBank (Table 3). The *B. tabaci* genotype groups were observed in varying frequencies across the Cameroon regions surveyed. SSA2 was the most predominant whitefly genotype group accounting for 35.6% of the whitefly isolates and also the most widespread occurring in three regions, namely Far North, North, and Adamawa. The second most predominant *B. tabaci* genotype group was SSA3 (22.2%) followed by SSA4 (13.3%) and MED (11.1%). The SSA1-SG1 was the whitefly genotype group least identified, accounting for 8.9% of the whitefly isolate and found only in the Adamawa region. The Adamawa region had the most whitefly genotype diversity with four (SSA1-SG1, SSA2, SSA3, and SSA4) of the five *B. tabaci* genotype groups identified from field samples in the country; *B. afer*, SSA2, and MED were identified in Far North and North regions, while SSA3 and SSA4 were found in the South–West region.

#### 3.1.3. Comparison of Whitefly Genotypes Found in Nigeria and Cameroon

Among the *B. tabaci* genotypes, SSA1-SG1, SSA2, SSA3, and MED were present in both countries. For these genotypes, there was a unique pattern of distribution. SSA2 and MED were observed to cluster in similar regions in both countries (Figure 4). These two genotypes were observed in Adamawa, Taraba, and Borno, which are North-eastern states in Nigeria that border Far North, North, and Adamawa in Cameroon—the same region where SSA2 and MED was found in Cameroon. In a similar pattern, SSA3 clustered in the lower part of the Nigeria–Cameroon border as SSA3 was observed in Benue and Cross River states in Nigeria which shares borders with the South–West region in Cameroon (Figure 4). There were also genotypes that were exclusive to each country. SSA1-SG3 was observed in Nigeria but not in Cameroon, while SSA4 and *B. afer* were observed in Cameroon and not in Nigeria.

### 3.2. Presence of CMBs in Identified Whitefly Species/Genotype Groups

ACMV was detected in 41.2% (40 out of 97) of the whiteflies screened in this study while EACMV was detected in 14.4% (14 out of 97) of the whiteflies (Table 4). Both single and mixed CMBs were detected.

In the Nigerian samples, single ACMV were detected in SSA1-SG1, SSA2, SSA3, and MED while single EACMV was detected in both SSA2 and MED. The MED genotype group was the only genotype that had mixed ACMV and EACMV detected.

In Cameroon, single ACMV was detected in at least one whitefly from each of the identified genotypes (SSA1-SG1, SSA2, SSA3, SSA4, MED, and *B. afer*). In contrast, SSA3 was the only genotype with single EACMV detected. Four genotypes (SSA1-SG1, SSA2, SSA3, and MED) had mixed ACMV and EACMV detected.

## 4. Discussion

Cassava mosaic disease (CMD) remains a major constraint to cassava production across Central and West Africa, while the continued westward spread of cassava brown streak disease (CBSD) presents an emerging threat to West Africa and previously unaffected Central African countries [5]. Given the role of whitefly vectors in the transmission of the causal viruses for both diseases, it is important to regularly assess the presence and distribution of whitefly species, especially along border regions which often serve as hotspots for disease incursion. This study provides information on the whitefly genotype present along the Nigerian–Cameroon border, which is the main border between the Central and West African cassava production zone. Using the mtCOI gene as a molecular maker, this study found that *B. tabaci* occurs widely on cassava plants across the border regions of Nigeria and Cameroon. This is consistent with previous studies that have been conducted in both Nigeria and Cameroon [21,31,33,34]. *B. tabaci* remains the most predominant whitefly across several regions, as previously reported in studies conducted across sub-Saharan Africa [17,21,37,43,44,45].

The second whitefly species identified in this study, the *B. afer,* was only recorded in Cameroon. *B. afer* is the second most abundant cassava-colonizing whitefly cryptic species in Africa [17,33]. Although *B. afer* was not observed in the Nigeria states assessed in the current study, it has been previously reported by Nwezeobi et al. [33] in Cross River state, which is one of the border states assessed in this study. Similarly, previous study by Kepngop et al. [34] has reported *B. afer* in South West, Cameroon which shares a border with Cross River, Nigeria. In this study, *B. afer* accounted for 8.9% of the whitefly isolates identified in the Cameroon border region. This low proportion supports earlier reports indicating that *B. afer* does not usually achieve sufficient levels of abundance to cause damage to the cassava plant and is therefore not considered a threat to cassava production [46,47]. While other whitefly species such as *T. vaporariorum, A. dispersus*, and *P. bondari* have been shown to colonize cassava [17], these species were not identified in this study.

*B. tabaci* have been grouped into cassava colonizing and non-cassava colonizing groups [9,25,48]. Amongst the non-cassava colonizing group, MED was found in cassava samples collected from Nigeria. MED is an invasive *B. tabaci* genotype group known for its high population and insecticide resistance [19]. This genotype group has been previously reported in several cassava-growing regions in Africa [49]. The MED genotype group accounted for 55.8% of the whitefly samples from Nigeria border regions. Although the MED genotype group has the ability to invade new regions and displace indigenous whitefly [44], we cannot explicitly conclude that the MED genotype group has replaced indigenous whitefly in Nigeria as this study covers only a proportion of the country. In addition, a study carried out by Nwezeobi et al. [33] reported MED as the least reported whitefly genotype group in eastern Nigeria. MED was also observed in Cameroon, and although not in high proportions as in Nigeria, it was observed to cluster in the upper border region in both countries. This clustering pattern suggests an agroecological preference of the MED whitefly and should be considered for future studies. The presence of MED poses a concern for whitefly control in the region, given that recent studies have shown the increased rate of insecticide resistance in MED [19,49].

The cassava-colonizing *B. tabaci* genotype groups identified in this study were: SSA1 (SG1, SG3), SSA2, SSA3, and SSA4. Among these, SSA2 was predominant in Cameroon while SSA3 was predominant in Nigeria. Both SSA2 and SSA3 have been reported in previous surveys from Nigeria, Benin, Cameroon, Togo, and Central Africa Republic [21,33,43,48,49]. Chen et al. [21] reported SSA2 as the most widely distributed cassava-colonizing *B. tabaci* genotype across sub-Saharan Africa and recorded it as comprising 44% of whitefly populations in Cameroon. Similarly, in this study, SSA2 made up 35% of the whiteflies identified in Cameroon, further buttressing its significant role in local CMD epidemiology. In contrast to this study and that of Chen et al. [21], Kepngop et al. [34] did not detect SSA2 in Cameroon, suggesting either sampling limitations or population shifts over time. In a similar pattern to the distribution of MED along the border regions, SSA2 clustered in the upper part of the Nigeria–Cameroon border while SSA3 clustered in the lower part of the border. The clustering of SSA3 in the lower part of the Nigeria-–Cameroon border is consistent with earlier work conducted by Nwezeobi et al. [33], where SSA3 was cited as the predominant whitefly in the eastern part of Nigeria. SSA3 was found in the western part of DRC that was not affected by the CMD pandemic and Namuddu et al. [44] suggested that SSA3 may have spread from West Africa to East Africa given that it was only just recently reported in Uganda.

Another important observation in this study was the low proportions of SSA1-SG1 (<9%) in both countries. A similar trend was previously reported in Cameroon [21,34]. Also, previous studies conducted in Nigeria also reported minimal presence of SSA1-SG1 [31,33]. In contrast, SSA1-SG1 has been reported as the most predominant genotype in most cassava-growing regions in East and Central Africa [9,50]. In the early 2000s, SSA1-SG1 was said to have replaced the SSA2 genotype group, which was predominant during the CMD pandemic in the 1990s [44]. Although SSA1-SG1 may have played an active role in the spread of cassava mosaic begomoviruses (CMBs) during the CMD pandemic, its limited presence in the current study location suggests that this genotype may not currently be the primary driver of CMD spread in the surveyed areas. This hypothesis can be further explored in future studies.

The other SSA1 subgroups identified in this study also occurred in low proportions. SSA1-SG3 was found in Nigeria but not in Cameroon. Similarly to this study, Kepngop et al. [34] did not report SSA1-SG3 in Cameroon. Other SSA1 sub-groups that were not recorded in this study include SSA1-SG2, SSA1-SG4, and SSA1-SG5. In contrast to this study, SSA1-SG5 has been identified in Nigeria and Cameroon in previous studies [21,33].

The presence of SSA4 in Cameroon, which is a Central African country, is consistent with what has been reported in previous studies. The study carried out by Chen et al. [21] suggested that SSA4 is restricted to Central Africa. Furthermore, Kepngop et al. [34] reported SSA4 as the most abundant whitefly collected on cassava plants in Cameroon. However, in this study, SSA4 was the third most abundant whitefly, accounting for 13% of whitefly identified in Cameroon. This is a pointer to the prevalence and widespread distribution of SSA4 in Cameroon.

In this study, ACMV was detected in *B. afer* from Cameroon. Although *B. afer* has previously been implicated in the transmission of cassava brown streak ipomoviruses (CBSIs). In the study conducted by Maruthi et al. [51], CBSIs transmission by *B. afer* was observed when the experiment was inoculated with *B. tabaci* and was not observed when *B. afer* was used alone. In addition, the efficiency of *B. afer* in transmitting CMBs has not been confirmed [52]. The detection of ACMV in *B. afer* in the present study does not necessarily imply vector competence or transmission capability, as the presence of viral DNA within a whitefly may indicate virus acquisition without successful transmission to a susceptible plant. Therefore, our findings should be interpreted as evidence of an association between *B. afer* and ACMV rather than confirmation that *B. afer* is a vector of ACMV or other CMBs. It would therefore be useful and informative to include *B. afer* in future CMBs transmission studies. In Nigeria, MED was the only genotype group that had both single and mixed detections of ACMV and EACMV. Although in minimal proportions, single ACMV detections, as well as mixed ACMV and EACMV, were also detected in the MED genotype group collected from cassava farms in Cameroon. This is particularly concerning since previous evidence had demonstrated that virus detection can increase the reproductive capacity of MED whiteflies, as demonstrated with tomato chlorosis virus (ToCV) [53]. However, further investigation is required to properly understand the relationship between CMBs and MED, especially in relation to the insect reproduction and virus transmission efficiency.

SSA1-SG1 also showed variable virus associations across both countries. In Nigeria, SSA1-SG1 was associated only with ACMV, while in Cameroon, single ACMV detections and mixed ACMV and EACMV were detected in isolates of this subgroup. Although the abundance of the SSA1-SG1 genotype was relatively low (<9%) in this study, it is still important to keep tracking their population in the study region due to their role in past CMD epidemics [36]. Only one SSA1-SG3 isolate was identified in Nigeria, and it tested negative for both viruses. SSA2, previously linked to severe CMD epidemics in East Africa, was found in both Nigeria and Cameroon. SSA2 samples tested positive for single ACMV and EACMV detections in Nigeria, whereas in Cameroon single ACMV detections and mixed ACMV and EACMV were detected. SSA3 displayed geographical variation in viral associations: single ACMV was detected in SSA3 isolates from Nigerian samples, while mixed ACMV and EACMV was detected in samples from Cameroon. SSA4, absent in Nigeria but present in Cameroon, was also associated with only ACMV.

While mitochondrial cytochrome c oxidase subunit I (mtCOI) sequencing remains a standard and effective marker for preliminary whitefly species identification and genetic grouping, recent genomic evidence [29] indicates that mtCOI alone may not always fully resolve certain sub-Saharan Africa (SSA) genotype groups. In addition, PCR assays were performed without technical replicates for individual samples, which may reduce confidence in the reproducibility of individual amplification results and means that occasional false-positive or false-negative reactions cannot be completely excluded. Nevertheless, the use of appropriate positive and negative controls, standardized DNA extraction and PCR procedures, and multiple independently collected biological samples provides reasonable confidence in the overall findings. Therefore, while the absence of technical PCR replicates represents a methodological limitation, it does not necessarily invalidate the observed patterns of whitefly genetic-group distribution and virus detection. Future studies incorporating technical PCR replicates, nuclear markers, or genome-wide single nucleotide polymorphisms (SNPs) will be valuable for independently validating the molecular findings and further resolving complex genetic relationships among *Bemisia* populations along the Nigeria–Cameroon border.

In summary, the results of the whitefly–virus association show the complex and dynamic nature of cassava virus epidemiology in Central and West Africa. The co-occurrence of multiple whitefly genotype groups, with mixed CMB presence, suggests the potential for overlapping transmission cycles and increased risk of mixed infections and recombination of viruses. Viral recombination has led to the emergence of virulent strains of CMBs in the past [7,12] and as such a robust virus disease management approach which addresses both the virus and vector is critical. This would include but not be limited to routine surveillance, increased awareness on the use of clean planting materials, removal of virus-infected plants, and deployment of resistant or tolerant cassava varieties [7,15,54].

## 5. Conclusions

The geographical distribution of the whitefly species is of great interest because of their importance as insect vectors for cassava viruses. This study provides critical information on the distribution of whitefly genotypes along the Nigerian–Cameroon border region. Five *B. tabaci* genotype groups were identified, including SSA1-SG1, SSA2, SSA3, and MED, which were observed in both countries, as well as SSA1-SG3 and SSA4 which were exclusive to Nigeria and Cameroon, respectively. This study also reports the presence of *B. afer* in Cameroon. The clustering of SSA2 and MED in the upper border regions, and SSA3 in the lower border regions of both countries, suggests potential agroecological preference of the whitefly genotype groups which could be influenced by climatic suitability or similarities in cassava cultivar usage. The distribution pattern also highlights the need for continued whitefly surveillance to inform effective regional management frameworks. This study also showed the presence of ACMV and EACMV both in single and mixed detections in multiple whitefly genotype groups across all regions, including *B. afer*. Overall, these findings provide essential baseline data to inform better management strategies of the vectors to reduce the spread and impact of these important viral diseases, which continue to be a threat to food security in major cassava growing regions.

## Figures and Tables

**Figure 1:**
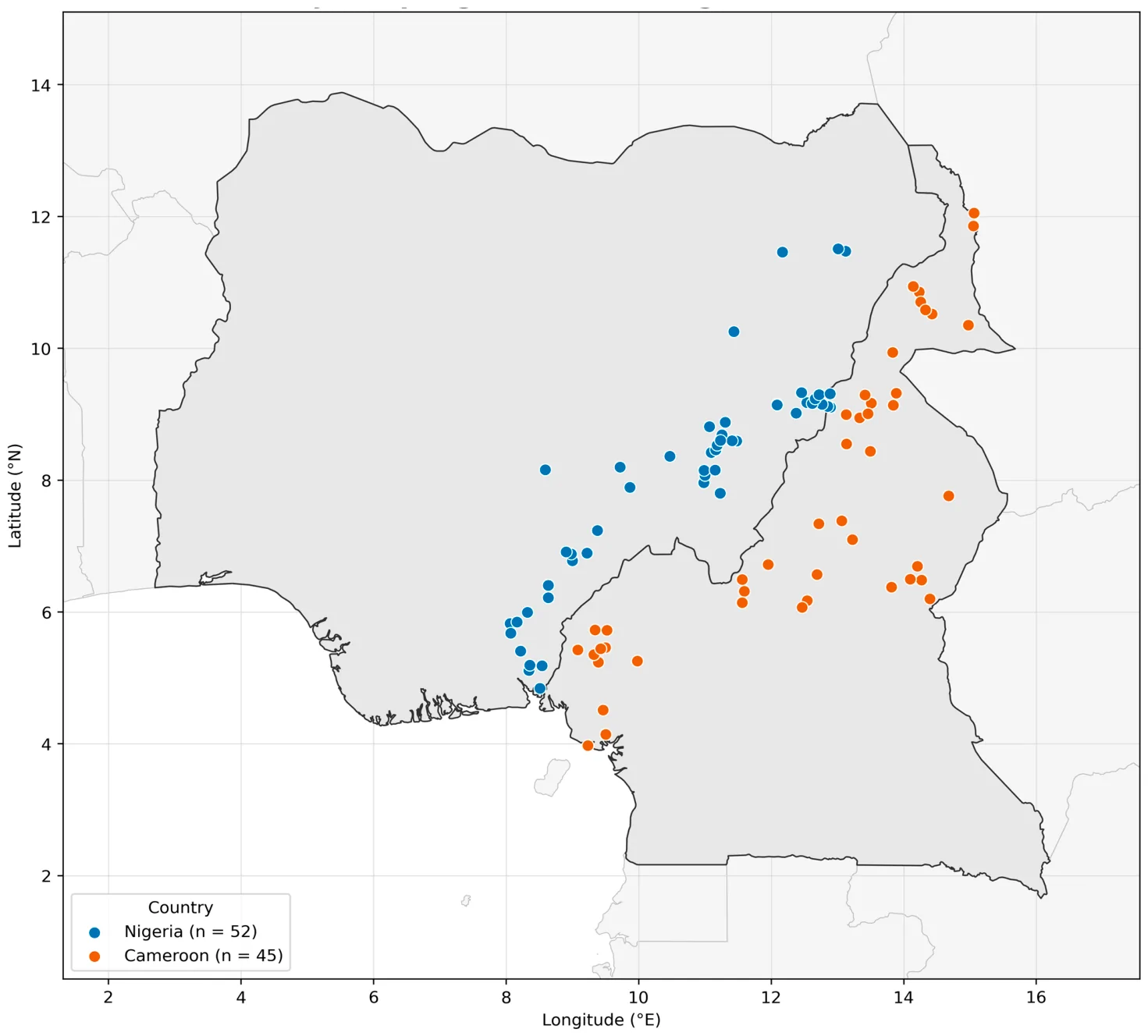
Whitefly sampling locations in Nigeria and Cameroon.

**Figure 2:**
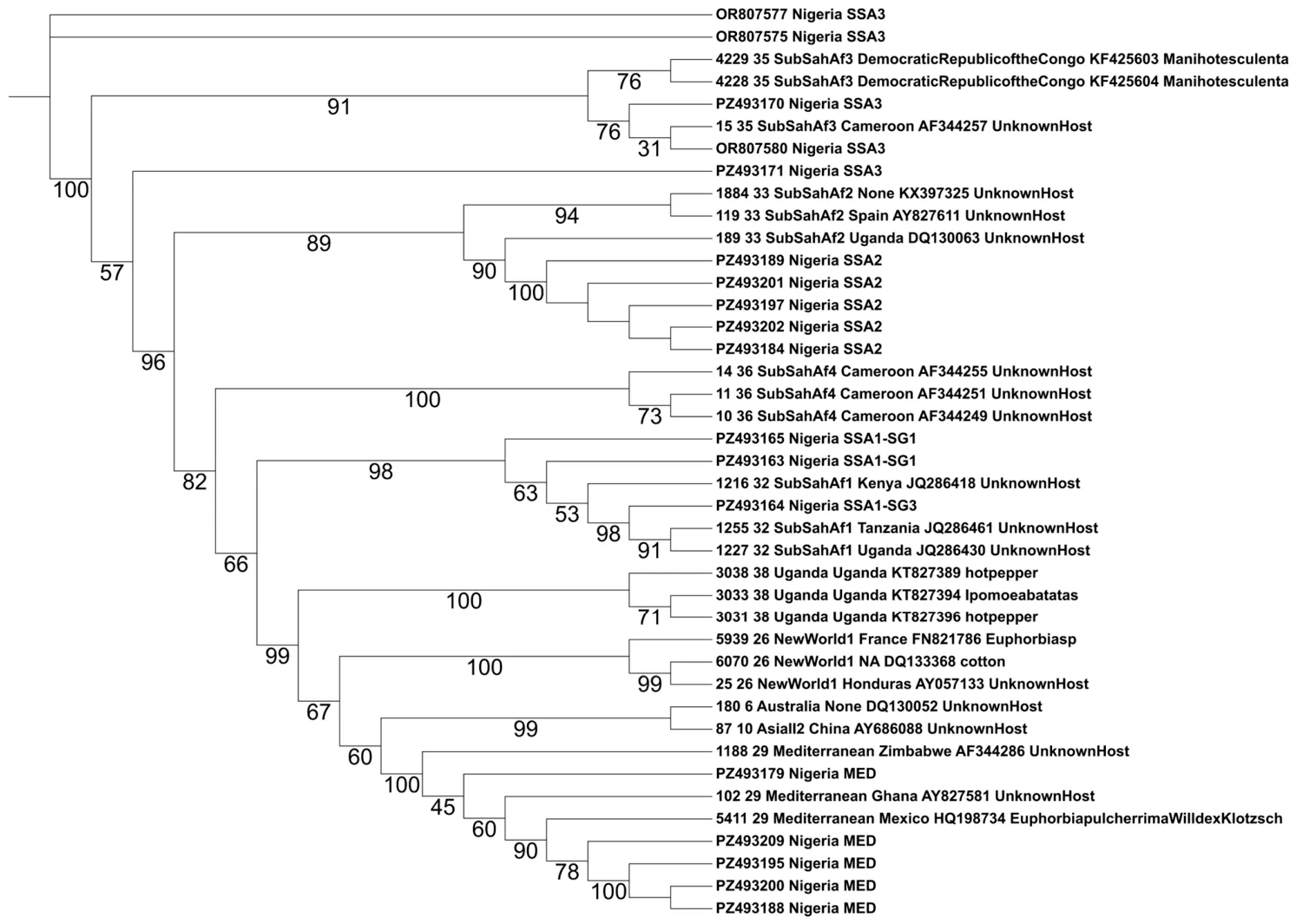
The phylogenetic tree showing the relationships between identified representative whitefly genotype groups from Nigeria and reference whitefly sequences from NCBI database.

**Figure 3:**
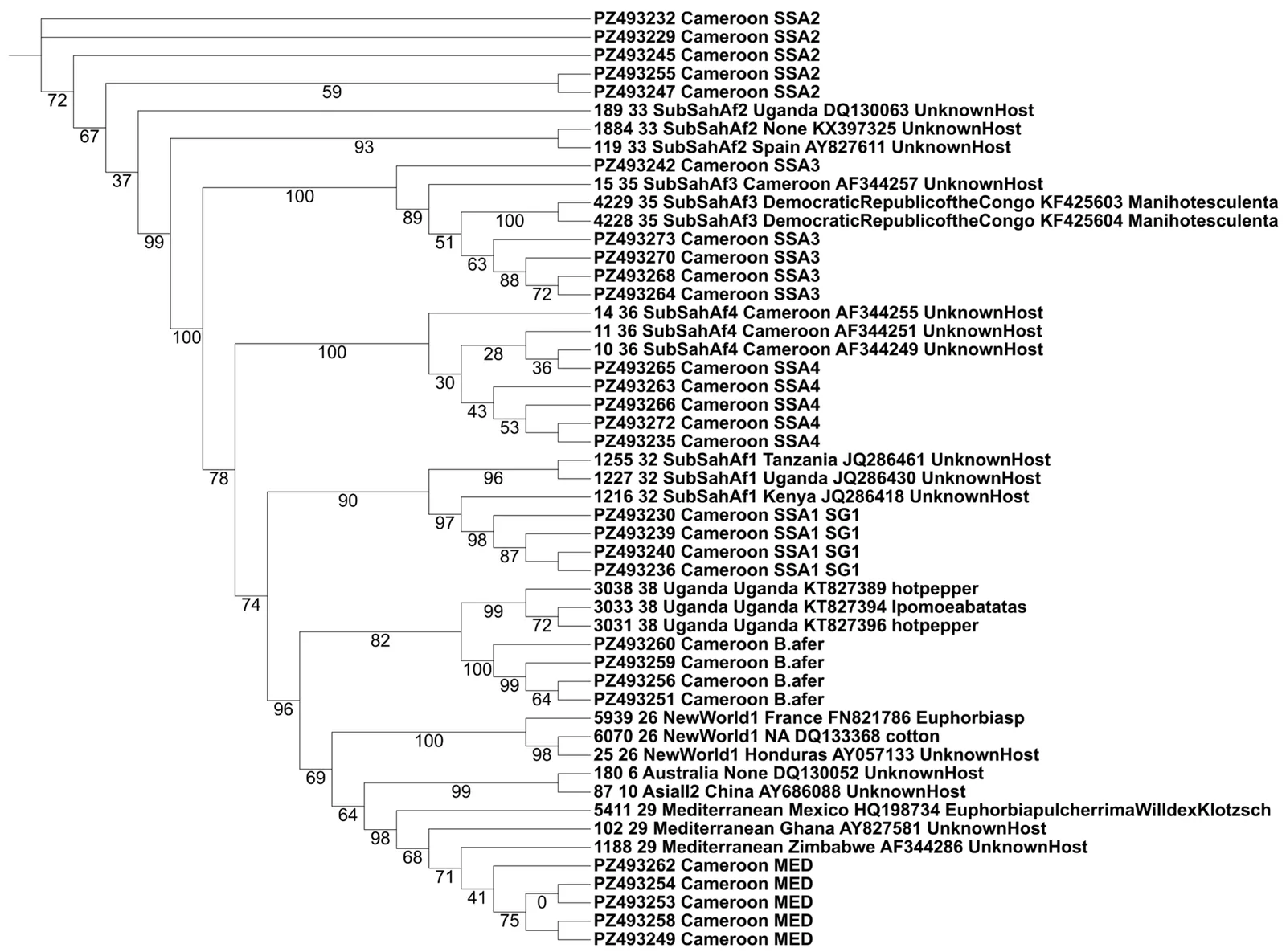
The phylogenetic tree showing the relationships between identified representative whitefly genotype groups from Cameroon and reference whitefly sequences from NCBI database.

**Figure 4:**
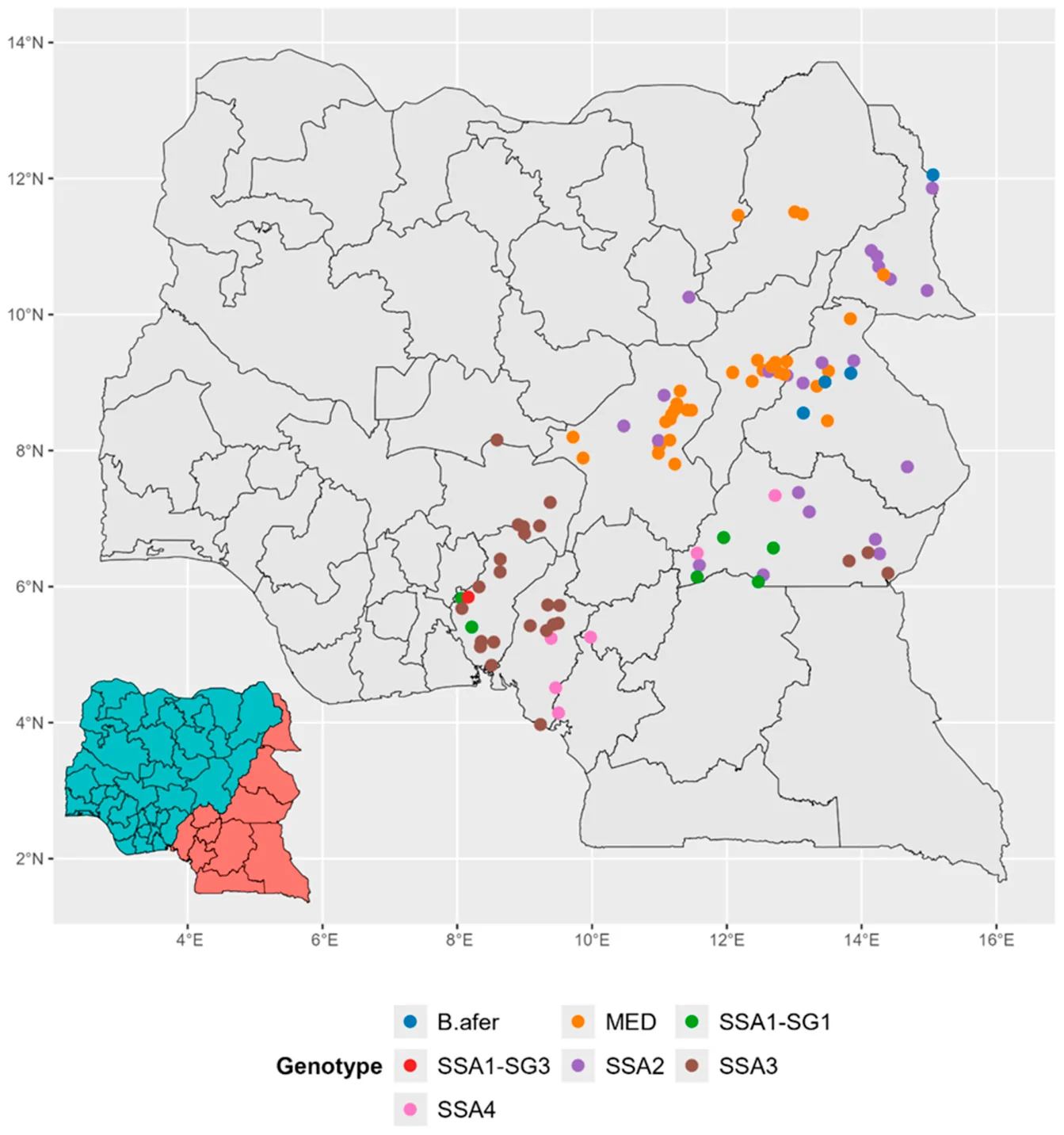
Distribution of whitefly species/genotype groups in cassava farms located in the border regions of Nigeria and Cameroon (borders between the Central and West African cassava production zone). The inset map highlights the relative geographic positioning of Nigeria (teal) and Cameroon (coral).

**Table 2:** Nucleotide similarities among whitefly (*B. tabaci*) sequences obtained from the Nigerian border using the primer pair 2195Bt and C012/Bt-sh2 to sequences obtained from NCBI GenBank.

Whitefly Genotype Group	Number of Sequences	Nucleotide Identity Similarity %	Accession Numbers	Countries
MED	29	96.69–100	FJ766429, AY903531, EU760758	Burkina Faso, Uganda, Cameroon
SSA1-SG1	2	99.56–100	HE573752, AY057180	DRC, Uganda
SSA1-SG3	1	99.42–99.85	JQ286457, HQ908649, AF418668	Tanzania, Benin, Ghana
SSA2	6	99.56–99.85	AY057202, AY827607, AY827604	Uganda, Nigeria, Mali
SSA3	14	99.1–99.7	KF425604, AF344257	DRC, Cameroon

**Table 3:** Nucleotide similarities among whitefly (*B. tabaci*) sequences obtained from the Cameroon border using the primer pair 2195Bt and C012/Bt-sh2 to sequences obtained from NCBI GenBank.

Whitefly Genotype	Number of Sequences	Nucleotide Identity Similarity %	Accession Numbers	Countries
*B. afer*	4	99.4–100	MZ333095	Kenya
MED	5	99.7–100	EU760758; AY903564; FJ766383	Cameroon; Uganda; Cote d’Ivoire
SSA1-SG1	4	99.5–100	JQ286484; HE573752; AY057195	Tanzania; DRC; Uganda
SSA2	16	97.7–100	AY057195; AY827604; AY827607	Uganda; Mali; Nigeria
SSA3	10	98.5–99.7	AF344257; KF425604	Cameroon; DRC
SSA4	6	99.5–100	AF344246;	Cameroon

**Table 4:** Proportion of identified whitefly species/genotype groups and presence of CMBs isolates across both countries surveyed.

Country		*B. afer*	MED	SSA1_SG1	SSA1_SG3	SSA2	SSA3	SSA4	Total
Nigeria	Number of isolates	0	29	2	1	6	14	0	52
	Proportion (%)	0	55.8	3.8	1.9	11.5	26.9	0	100
	Single ACMV	0	10	1	0	2	5	0	18
	Single EACMV	0	4	0	0	2	0	0	6
	Mixed ACMV + and EACMV detection	0	2	0	0	0	0	0	2
Cameroon	Number of isolates	4	5	4	0	16	10	6	45
	Proportion (%)	8.9	11.1	8.9	0	35.6	22.2	13.3	100
	Single ACMV	2	1	2	0	3	4	3	15
	Single EACMV	0	0	0	0	0	1	0	1
	Mixed ACMV + and EACMV detection	0	1	1	0	3	1	0	6

## Data Availability

Partial mtCO1 sequences of the *B. tabaci* whiteflies obtained in this study were deposited in GenBank under accession numbers PZ493163-PZ493174; PZ493176-PZ493210; PZ493229-PZ493273; OR807575; OR807577; and OR807579-OR807581.

## Abbreviations

The following abbreviations are used in this manuscript:

ACMV	African cassava mosaic virus
AFLP	Amplified Fragment Length Polymorphism
ASL	African Silver-leafing
BLAST	Basic Local Alignment Search Tool
CAPS	Cleaved Amplified Polymorphic Sequences
CBSD	Cassava brown streak disease
CBSIs	Cassava brown streak ipomoviruses
CMBs	Cassava mosaic begomoviruses
CMD	Cassava mosaic disease
DNA	Deoxyribonucleic acid
EACMV	East African cassava mosaic virus
EACMV-UG	East African cassava mosaic virus- Ugandan variant
GPS	Global Positioning System
kbp	kilobase pair
MEAM	Middle East–Asia Minor
MED	Mediterranean
mtCOI	Mitochondrial cytochrome oxidase 1
NCBI	National Center for Biotechnology Information
nt	Nucleotide
PCR	Polymerase chain reaction
RAPD	Random amplified polymorphic DNA
SCAR	Sequence characterized amplified region
SG	Sub-group
SSA	Sub-Saharan Africa
UV	Ultraviolet
